# Elucidation of potential challenges and prospects for regional tuberculosis interventions in East and Horn of Africa: a cross-sectional program assessment

**DOI:** 10.11604/pamj.2021.39.279.28028

**Published:** 2021-08-27

**Authors:** Anthony Martin Toroitich, Workneh Gebeyehu, Fatuma Ibrahim Adan, Christine Ogola, Hassan Muktar Mohamed, Victor Ombeka, Charles Ogolla, Shadrack Oiye

**Affiliations:** 1 Intergovernmental Authority on Development, Djibouti, Djibouti,; 2 United States Agency for International Development (USAID) Kenya and East Africa, Health Population and Nutrition Office, Nairobi, Kenya,; 3 The Royal Netherlands Tuberculosis Foundation, Tuberculosis Foundation, The Hague, Netherlands

**Keywords:** Tuberculosis interventions, cross-border areas, challenges, prospects

## Abstract

**Introduction:**

cross-border mobility of persons with Tuberculosis (TB) is a global public health concern. We aimed at documenting health systems´ potential bottlenecks and opportunities in pulmonary TB continuum of care in cross-border expanses of East and Horn of Africa.

**Methods:**

a cross-sectional program assessment with descriptive analysis of TB services, health staff capacities, diagnostic capacities, data management and reporting, and treatment outcomes. Data were extracted from health facility TB registers and semi-structured key informant interviews conducted in selected 26 cross-border sites within the 7 member states of the Intergovernmental Authority on Development (IGAD) region.

**Results:**

the overall cross-border TB cure rate in the year preceding the study (37%) was way beneath the global target with considerable variations amongst the study countries. The restricted support to the cross-border health facilities was mediated and even exacerbated by expansive distances from the respective capital cities. Restricted geographical access to the facilities by cross-border populations was a longstanding challenge. Substantial staffing gaps, TB service delivery capacity needs and inadequate diagnostics were noticeable. The TB control guidelines were not harmonized between the countries and the inter-country referral systems were either absent or inappreciable, contributing to ineffective cross-border referrals and transfers. The frail linkages between stakeholders were contemptible, but increasing governments´ commitments in tackling infectious diseases were encouraging.

**Conclusion:**

cross-border TB interventions should drive regional TB policies, strategies and programs that sustain countries´ coordination, harmonization of management guidelines, advocacy for increased human resources support, enhanced capacity building of cross-border TB staff, adequate diagnostics equipping of the cross-border health facilities and seamless transfer and referral of patients traversing boundaries.

## Introduction

Infectious diseases remain a major global concern and control entails concerted efforts nationally and regionally. Pulmonary Tuberculosis (TB) disease is almost exclusively airborne, preventable and is a curable disease. It is caused by the bacteria *Mycobacterium tuberculosis* and is of high public health importance. It has a fatality rate of 45% among HIV-uninfected and almost 100% among the HIV-infected [1]. About 1.4 million people succumbed to TB in 2019, including 208,000 people with HIV [1]. In sub-Sahara Africa, the tuberculosis epidemic is on the rise due to several factors, the most imperative being the HIV epidemic with HIV co-infected accounting for over half of the TB cases [2, 3]. Health systems in Africa continue to grapple with refining preventive measures, diagnostics laboratories and infrastructures, case detection, treatment, recording and reporting systems. The functionalities of these systems are so deprived that defining the actual extent of the TB problem is problematic [2]. Further, with inappropriate use of anti-TB medicines, poor-quality drugs and premature ceasing of treatment, the hard-to-treat multidrug-resistant TB is becoming more common [1].

Cross-border mobility of diseases not only affects the regional economy but also carries obvious health risks that can compromise the health of millions of people in the region and jeopardize treatment [4]. Recent poliovirus (cVDPV2) outbreak in the Horn of Africa [5], and the ongoing coronavirus pandemic are clear examples of how communicable diseases can spread across borders. The two associated illnesses of TB and HIV in East and Horn of Africa are also complicated by human cross-border movements which cannot be halted due to the human right to freedom of movement; and the right to health demands that the health challenges presented by these movements are addressed [6, 7].

Global TB strategies aim at reaching at least 90% of TB-infected and a minimum of 90% treatment success [8]. Subtle but pertinent dissimilarities in the countries´ TB control measures exist and this compromises the effectiveness of the continuum of care in the cross-border areas. Regional efforts in addressing the health threats due to TB are therefore pertinent and increasingly becoming well recognized in sub-Sahara Africa [9].

The Intergovernmental Authority on Development (IGAD) is one of the regional economic communities (REC) of the African Union (AU) established in 1996 in the Horn of Africa, Nile Valley, and the African Great Lakes Region. IGAD member states are Djibouti, Ethiopia, Kenya, South Sudan, Somalia Sudan and Uganda. One of the key regional public health priorities of IGAD is to establish and strengthen resilient health systems in the marginalized cross-border areas which hitherto remain neglected. About 20% of IGAD population lives in these areas. IGAD describes Cross-border Mobile Populations (CBMPs) as people living in cross-border areas and includes mobile pastoralists in search for pasture, seasonal cross-border labourers, economically active persons, undocumented migrants, and internally displaced persons and their host communities. These groups are relatively more vulnerable and thus need equitable and more resilient quality healthcare services, and intentional and focused efforts must be put in place to target them [7].

To inform the scale-up of regional pulmonary TB control efforts and in the cross-border areas of East and Horn of Africa, there was a need to generate evidence for potential cardinal considerations in regional and cross-border policies, strategies and programs. It is for this reason that IGAD in partnership with USAID and Challenge TB (CTB) coordinated a regional cross-border health system assessment aimed at assessing the challenges faced and presenting opportunities in the TB control among the cross-border mobile populations.

## Methods

**Study design and general approach:** this was a cross-sectional program assessment of a sample of IGAD region cross-border sites using a descriptive-analytical approach. The study was conducted between April and July 2018 and entailed desk review of data and information at the selected health facilities and key informants´ interviews with the health facility staff as well as the National TB Prevention and Care Program (NTP) managers. Quantitative and qualitative data were collected on access to TB services, health staff capacity, diagnostic capacity of health facilities, data management and reporting and treatment outcomes. Data and information analyzed descriptively and triangulated to take inventory of challenges and opportunities in TB diagnostics, treatments and attendant support mechanisms. A cross-border area was defined as a geographical area made of several local authorities that are co-located yet belong to different countries and in a position to augment their competitiveness in health care.

**Figure 1:**
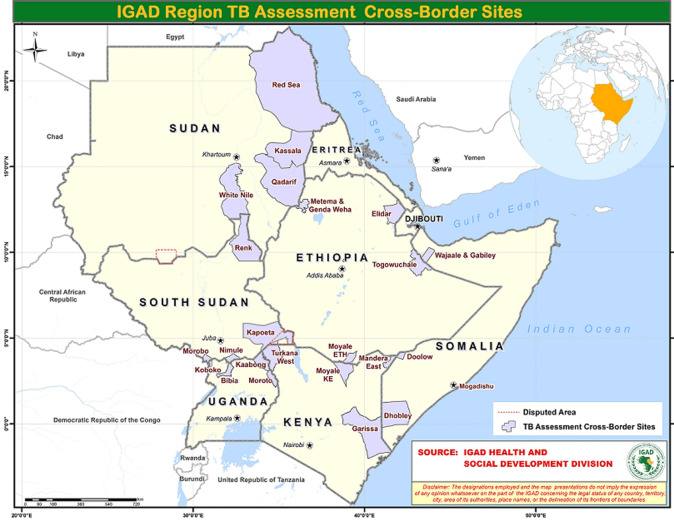
sampled IGAD region cross-border sites for the TB assessment

**Study area and participants:** a sample of 26 cross-border districts and counties (depending on the country's sub-national jurisdictions) accounted for 22% of all cross-border sites in the IGAD region. These areas, shown in Figure 1, were selected as priority IGAD health programs sites and for the planned cross-border TB interventions. The IGAD health program matrix of selecting priority cross-border areas considered the then relative levels of the key health outcomes, human population traffic and the distances from both the capital cities and major regional towns. This selection criterion was thus inclined towards areas with the propensity for greater absolute numbers of TB patients and relatively more underprivileged in health service access. As shown in Figure 1, Ethiopia and Somalia contributed 5 sites each. From each of the remaining countries, 4 sites were drawn.

All the 116 Cross-border Health facilities (CBHFs) in the selected areas were considered and included 28 hospitals, 59 health centers, 16 health posts/dispensaries, 4 private medical clinics and 9 primary health care units. Key informants from each IGAD member state were the NTP managers, NTP monitoring and evaluation (M and E) focal points, nurse supervisors in the cross-border and TB focal points in CBHFs.

**Data sets collected and tools used:** using semi-structured tools, key informants provided information on the types of CBMPs in the respective cross-border sites, movement patterns, time taken to walk from the health facilities to the furthest villages (to indicate access to TB services), health staff capacity to support TB services provided by NTP managers to the health facilities. The same tools were used to extract information on the existence of functional diagnostics equipment at the facilities, TB data management and reporting and challenges experienced in TB diagnostics and treatment.

Data on TB outcomes were collected from the various TB registers as were provided by the TB health facility focal points. A year preceding April 2018 data were collected on Upper Respiratory Tract Infections (URTI), TB cases notified, MDR cases and TB treatment outcomes (treatment completion, deaths, transfers, and cured). Using a checklist, information was also drawn on the health facility staff, TB services provided and TB recording and reporting tools.

**Data handling and analysis:** quantitative data were entered into MS Excel and analyzed in Statistical Package for Social Scientists (SPSS) version 20 for descriptive analyses. For each of the guide key informants' questions, responses were classified into all possible categories. All the guide questions and summarized responses in categories were then organized as per the themes of the movement pattern, CBMP types, challenges, and opportunities. Interpretations in these themes allowed for the triangulation of data/information generated from different sources in the respective cross-border sites.

**Study approval and ethical considerations:** this study did not involve humans´ subjects or invasive procedures. Permission was sort from all the IGAD member states' NTPs where each of the respective managers reviewed the study protocol before approval. While extracting data from the records, names of TB patients were blinded and in no case were references made to them. Key informants 'interviews were held in private soundproof places and confidentiality was assured. Permission from all the informants was obtained before commencing the interviews.

## Results

**CBMP inter-county movement pattern and access to health services:** the distances of the sampled cross-border areas from their respective capital cities range from <200Km in South Sudan to >700km in Ethiopia. Table 1 depicts by country, the cross-border movement pattern, types of CBPM and approximated mean time taken to the health facilities from furthest villages. The sampled cross-border areas of Kenya had the highest number of inter-country population movements, and in Ethiopia, those on the move were mainly not from other countries. The categorization of CBPMs indicates that major economic activities were nomadic pastoralism and various cross-border trades. Kenya (Turkana west and Dadaab) and Somalia (Dhobley and Dollow) had refugees and returnees due to the conflict situation in Somalia and South Sudan respectively. On average, more time was taken walking from the furthest villages to health facilities in Uganda and Ethiopia (11 hours) and (13 hours) respectively. The furthest villages were relatively closer in South Sudan and Sudan CBHFs.

**Table 1:** movement pattern, category of CBMP and time taken to the health facilities

Country (number of HFs sampled)	Movement pattern (#of countries of origin	Time taken (hrs) walk to the HF from the furthest villages ¥	Types of CBPM					
			Nomads and Pastoralists	Cross border traders	Truck drivers	Commercial sex workers	Casual workers	Refugees and returnees
Ethiopia (n= 19)	0^Ω^	10.6	✓	✓	✓	✓	✓	✘
Kenya (n= 25)	5	7.2	✓	✓	✘	✘	✘	✓
Uganda (n= 19)	3	12.5	✘	✓	✘	✓	✓	✘
Somalia (n= 17)	2	7.5	✓	✓	✘	✘	✘	✓
South Sudan (n= 18)	2	4.3	✓	✓	✘	✘	✘	✘
Sudan (n= 20)	3	5.6	✓	✓	✘	✘	✘	✘

CBMP=Cross-border Mobile Population, HF=Health Facilities

✓= Yes, ✘= No

¥, hrs= hours. The mean hour taken to a health facility walking from the furthest villages

Ω Those on move originated from the same country (Ethiopia)

**Health worker staffing, TB diagnostics and reporting:** Table 2 shows the staffing, available TB diagnostic services and TB record keeping in the CBHFs. Health worker staffing gaps differed from country to country with the highest established filled positions being laboratory technicians, nurses and pharmaceutical personnel who were in 70%, 60% and 60% respectively in the sampled CBHFs. The lowest established filled posts were medical officer and clinician/health officer positions, in 49% and 52% facilities respectively. CBHFs in Kenya, Ethiopia and Somali borders lacked medical officers (zero), while those in Uganda, Ethiopia, Sudan, and South Sudan lacked laboratory technicians (data not shown in Table 2). It was apparent from the key informants´ interviews that the nurses were the most critical inpatient follow-up, evaluation of treatment outcomes and providing a link to the local community, in addition to being the facility in-charge.

**Table 2:** staffing, TB diagnostic services and record keeping in CBHFs

Category	% of facilities (n=118)
Staffing	% having positions filled
Medical officer	49.1
Clinicians/Health Officers	52.1
Nurses	60.2
Pharmaceutical personnel	60.2
Lab technicians	69.5
**TB services**	**% offering TB services**
TB counseling and testing	67.5
TB referral	70.1
First line TB drugs	57.3
Second line TB drugs	23.1
TB treatment	58.1
TB diagnosis	48.7
ART services	42.7
Acid-fast Bacilli (AFB) smear microscopy	41.9
Gene Xpert	17.9
X-ray services	13.7
Culture for diagnosis	6.8
Drug susceptibility testing (DST)	6.0
**TB recording and reporting tools**	**% utilizing presumptive registers**
Presumptive TB registers	45.7
TB referral and counter referral slip/forms	52.6
Lab TB registers	54.3
TB registers	56.9
OPD registers	90.5
Monthly reporting forms	91.4

CBHFs=Cross-border Health Facilities

It was reported that outpatient services were popular for various illnesses including upper respiratory tract infections, TB, sexually transmitted infections, immunization, family planning, antenatal clinical services, malaria, typhoid, injuries and other communicable diseases. As shown in Table 2, the majority of the sampled CBHFs were offering TB counselling and testing, TB referral services, had first-line TB drugs. About half (49%) of the CBHFs were offering TB diagnostics, while 43%, HIV treatment services, and 42% the AFB microscopy. Gene Xperts were available only in about 18% of the CBHFs, and together with the limited availability of TB microscopy and X-ray services, the capacity to detect multidrug resistance TB was suboptimal. It was also gathered anecdotally that X-ray machines, microscopes, and GeneXpert functionalities were not guaranteed due to lack of regular service and calibration.

As shown in Table 2, most CBHFs (91%) had monthly reporting forms and captured patient details in the outpatient department (OPD). registers The OPD registers did not disaggregate patients by the population. About 45% of facilities were utilizing TB recording/reporting tools with very low utilization being observed in some countries - as low as 5% in Sudan. All diagnostic facilities had TB OPD registers, but roughly half had presumptive, referral and counter referral slips, laboratory TB, and TB registers. Only 30% of facilities in Sudan border areas assessed had TB referral forms, Somalia and South Sudan 35% each, Ethiopia at 61%, Kenya at 65% while Uganda was at 79% (not shown in Table 2). Only Kenya and Uganda were using a referral form developed by the East African Community (EAC) for effective and efficient transfer and referral of clients across the national borders.

**Challenges and opportunities from key informants interviews:** key informants´ interviews with the NTP managers and M&E focal points, nurse supervisors and health facility TB focal points indicated that most TB patients were being referred or transferred to other health facilities across the borders using a letter scribbled by a healthcare provider who did not see the patient again or even got feedback on patient treatments progress or outcomes. The referrals were necessitated by lack of diagnostics and treatment in the originating facilities. Transfers were mainly for the reasons of migration or patient return to the native country. There were no mechanisms for feedback after patient referrals and transfers leading to inaccurate documentation on treatment outcomes except in Turkana/Moroto border areas. There existed no formal or non-formal TB cross border health committee to enhance communication amongst the facilities. Even the non-formal committee in Turkana/Moroto did not meet regularly. The language barrier was also a widespread challenge as depicted by the Amharic language (Ethiopia), French (Djibouti) and the Arabic (Sudan/South Sudan) and English in the corresponding bordering states. Each CBHFs used TB management protocols developed by their respective ministries of health that were not harmonized and aligned to current WHO recommendations thus making regional management of patients difficult. KIIs in the Kenya/Ethiopia border areas indicated that the use of different calendar years by Ethiopia and Kenya made it difficult to distinguish dates.

Across all the border areas, support supervision from the regional or national level remained a challenge with minimal contact with their respective NTPs. It was also reported that there were no regular TB related training targeting healthcare providers. There was a sense of CBHFs being generally neglected with limited budgetary allocation to support cross-border healthcare provision.

There was generally no formal linkage existing between CBHFs and immigration or border control services to facilitate the quick entry of TB patients, tracking their movements and support them to access care. However, in Uganda, there were formal arrangements for TB management meetings and the immigration officials were invited to participate. Albeit not formally, Somalia health facilities contacted the immigration department to facilitate cross border referrals. Some border areas such as Turkana West (Kenya) collaborated with international organizations like the United Nations High Commissioner for Refugees (UNHCR) and the International Organization for Migration (IOM) for referral and tracking of TB patients. Most NGOs had other health and nutrition activities in the geographical catchment areas for potential support to TB diagnostics, treatment and follow-ups.

**TB care outcomes:** most facilities reported data through the National Health Information Management Systems (HMIS) including the District Health Information System 2 (DHIS 2). Table 3 shows the TB diagnostic and treatment outcomes year preceding the study. Out of all the notified TB cases, ~20% were CBMP (nomads/pastoralists/refugees). Uganda (84%) reported the highest proportion of TB patients who were CBMP and was followed by Kenya (32%) and Sudan (25). Those treated in foreign countries accounted for 8.5% of all cases and were disproportionately high in Kenya (14%). Successful referral to other countries was reported at 0.7%, highest in Kenya and Uganda, all at 2%. About half (49%) of the notified TB cases completed treatment. About 37% were cured way below the target of 90% target with only Uganda close to achieving the target, shy by 6%. The lowest cure rate was observed in South Sudan (10%).

**Table 3:** TB treatment outcomes in 26 cross-border areas in the 118 health facilities assessed

Indicator	Ethiopia	Kenya	Somalia	South Sudan	Sudan	Uganda	Total
**TB diagnosis and referral in the past one year**							
Number of upper respiratory tract infection (URTI) cases	13,027	195,093	16,695	3,074	34,118	60,011	322,018
Number of Presumptive TB Cases in cross-border areas	267	5,642	4,888	3,154	3,154	862	**16,680**
Number of TB cases notified	1,314	1485	1452	576	466	316	5,609
Number of CBMP (nomads/ pastoralists/refugees) detected with TB	91 (6.9)	481 (32.4)	163 (11.2)	DNA	121 (26.0)	264 (83.5)	**1120 (20.0)**
Number (%) of MDR cases among CBMP	6 (0.5)	9 (0.6)	19 (1.3)	9 (1.5)	4 (0.9)	4 (1.3)	**51(0.9)**
Number (%) of CBMP TB patients attended to from other country	3 (3.3)	67 (13.9)	6 (3.7)	6	1 (0.8)	8 (3.0)	**91 (8.5)**
Number (%) of TB patients successfully referred to the other country	0 (0)	10 (2.0)	3 (1.8)	5	0 (0)	5 (1.9)	**23(0.7)**
**TB treatment outcomes**							
Number (%) of TB patients cured	356 (27.1)	506 (34.1)	764 (52.6)	60 (10.4)	132 (28.3)	266 (84.2)	2,084 (37.1)
Number (%) completed treatment	364 (27.7)	585 (39.4)	777(53.5)	222 (38.5)	332 (38.5)	483 (152.8)	2,763(49.2)
Number (%) of TB patients´ dead	10 (0.8)	42(2.8)	51 (3.5)	15 (2.6)	19 (4.0)	24 (7.6)	161 (2.9)
Number (%) of TB cases loss to follow up	1 (0.1)	39(0.2)	30 (2.1	63 (10.9	33 (7.1	191 (6.0	357 (6.4)
Number (%) transferred out	4 (0.3)	1 (0.1)	56 (3.9)	17 (3.0)	7 (1.5)	90 (28.5)	175 (3.1)

CBMP=Cross-border Mobile Population; MDR= Multi-Drug Resistant

## Discussion

We had aimed at documenting health systems´ potential bottlenecks and prospects in pulmonary TB continuum of care in cross-border areas of East and Horn of Africa. The challenges we reported were partly implicated in the overall below-target TB treatment completion and sub-optimal cure rates among cross-border mobile populations. These challenges emerged from the general neglect by the health systems, geographical remoteness, human resources limitations, general unavailability of TB diagnostics, ineffective regional (and inter-country) collaborations, in-vain inter-facility linkages, and variation in national TB treatment guidelines. Prospects for improving TB diagnostics and treatment existed in IGAD member states' political and health systems´ commitments in responding to infectious diseases, the sound and best practices demonstrated by some collaborating countries, emerging convening powers of intergovernmental organizations and their visibility in supporting cross-border health.

The expansive distances separating cross-border areas and their respective capital cities, and long hours of travel by CBMPs to access health services is a fundamental geographical challenge. Challenges facing these facilities are aggravated by the geographical distances from the capital cities - thus increasing the risk of being ‘forgotten´. In sub-Saharan Africa, most healthcare systems are managed centrally and this is largely thought not to be viable in the effective delivery of health service in the hard-to-reach areas [10]. Even in the devolved or partly devolved systems, the general neglects of the cross-border areas as depicted by limited resource provision and sub-optimal support supervision are still experienced owing to the ineffectiveness of both national and sub-national health systems. The limited and intermittent resource support [11] may explain the inadequate TB diagnostics equipment including Gene Xperts which were rare, impacting negatively on access to testing. In these resource-constrained countries (including in IGAD region), support supervision is an integral part of health service delivery but is not effective [12]. Among the CBMPs, access to TB and other health services is hampered by the long travel distances given that the road networks in these areas are mostly in disrepair. The long travel distances negatively impact referral and transfer of patients. There is also a delay in the conveyance of samples to regional health facilities leading to the increased turnaround [13, 14].

Just like for other healthcare services, fewer than needed health care providers compromise TB diagnosis, treatment, and care. These areas are more vulnerable to staff attrition. Health staffing gaps were apparent and much more for the nurses who were relatively more critical in providing treatment, patient follow-up, evaluation of treatment outcomes and providing a link to the local community. The laboratory staffs were generally more available compared to other cadres, but with lack of diagnostics equipment, their value in TB care was diminished. The need to provide sufficient human resources and support human capacity development in CBHFs was evident, to enable the effective provision of essential TB care services. Most cross-border areas are remote and health care staff turnover is disproportionately higher due to the harsh living conditions experienced [15, 16]. CBHFs staff also require regular support supervision from national, sub-national and regional levels, and this must be together with regular and relevant TB-related training.

As at the time of this present study, TB treatment guidelines in the IGAD region had not been harmonized. The variations in some country calendars and official languages further complicated the synchronization of the treatment guidelines across the borders. Harmonization challenge is not peculiar to the East and Horn of Africa only. The need for having matching guidelines has also been emphasized for Southern and Western Africa countries [17]. Further, lack of harmonized documentation practices had the propensity to negatively impact the continuum of care across the border owing to the need for effective patient follow-up. Informal cross-border referrals and transfers were ineffective and there was limited feedback to the originating facilities. Harmonization of guidelines, reporting and referrals forms was hampered by a general lack of intentional collaborative efforts between the countries, health facilities and other key stakeholders. Sharp variations in the TB outcomes among the countries were an indication that countries need to learn lessons and adopt best practices from each other.

Opportunities to improve TB care services include the existing political and administrative commitments for cooperation at cross-border areas. These commitments have been more elaborated by the Covid-19 pandemic where cross-border collaborations are inevitable. The willingness of heads of NTPs to form a regional TB forum to address cross-border issues is an imperative opportunity. The presence of IGAD, East African Community and East, Central and Southern African Health Community (ECSA-HC) as health-focused regional intergovernmental organizations is also an opportunity to improve coordination and support for current and arising transboundary issues.

Availability of ART and HIV/AIDS counselling and testing services at most of the border facilities offers an opportunity for integration of TB/HIV activities thereby increasing case finding and improved treatment outcomes. The Kenyan model of operationalization of TB manyatta (resident facilities near health facilities) can regionally be adopted to restrain the movement of the mobile population during treatment.

The existing unstructured linkages between facilities across the border can be formalized and streamlined. There is an apparent need for countries to establish functional cross-border health committees whose mandate will not only be to restructure communication amongst the border area health authorities but also strengthen the connections between transboundary health facilities. There is also the opportunity of leveraging on the presence of UN agencies and other development partners working in cross-border areas to strengthen TB capacity.

This study was not without limitations. The sampling of 26 out of the current 118 possible sites was based on the wider IGAD health program priority cross-border areas. Interpretations here-in presented are therefore restricted only to the sampled sites and extrapolations for other sites must be done cautiously.

## Conclusion

The implications of the findings are potentially relevant to health-focused intergovernmental organizations in the East and Horn of Africa, including IGAD, East African Community (EAC) and East, Central and Southern African Health Community (ECSA-HC). The general neglect of cross-border health facilities is a critical persisting hindrance to achieving optimum TB treatment outcomes. Intergovernmental (regional) organizations are hitherto gaining heightened visibility, reputation and member states´ convening powers, and can leverage on the member states´ increasing commitments in controlling communicable diseases. The prevailing but restrained best practices in cross-border TB care can be further demonstrated and scaled up. Regional and cross-border TB interventions need to consider policies, strategies and programs that support improvement in inter-country collaboration, harmonization of TB management guidelines, advocacy for adequate human resources, support for health staff capacity building, satisfactory diagnostics equipping and seamless transfer and referral of patients across national borders.
